# The Synthesis and Assembly Mechanism of Micro/Nano-Sized Polystyrene Spheres and Their Application in Subwavelength Structures

**DOI:** 10.3390/mi15070841

**Published:** 2024-06-28

**Authors:** Yeeu-Chang Lee, Hsu-Kang Wu, Yu-Zhong Peng, Wei-Chun Chen

**Affiliations:** 1 Department of Mechanical Engineering, Chung Yuan Christian University, Chung Li 32023, Taiwan; pudding1214@hotmail.com (H.-K.W.); al950808@gmail.com (Y.-Z.P.); 2 National Applied Research Laboratories, Taiwan Instrument Research Institute (TIRI), Hsinchu 30076, Taiwan; weichun@narlabs.org.tw

**Keywords:** colloidal lithography, polystyrene (PS) spheres, floating assembly method, subwavelength structures

## Abstract

The following study involved the utilization of dispersion polymerization to synthesize micron/nano-sized polystyrene (PS) spheres, which were then deposited onto a silicon substrate using the floating assembly method to form a long-range monolayer. Subsequently, dry etching techniques were utilized to create subwavelength structures. The adjustment of the stabilizer polyvinylpyrrolidone (PVP), together with changes in the monomer concentration, yielded PS spheres ranging from 500 nm to 5.6 μm in diameter. These PS spheres were suspended in a mixture of alcohol and deionized water before being arranged using the floating assembly method. The resulting tightly packed particle arrangement is attributed to van der Waals forces, Coulomb electrostatic forces between the PS spheres, and surface tension effects. The interplay of these forces was analyzed to comprehend the resulting structure. Dry etching, utilizing the PS spheres as masks, enabled the exploration of the effects of etching parameters on the resultant structures. Unlike traditional dry etching methods controlling RF power and etching gases, in the present study, we focused on adjusting the oxygen flow rate to achieve cylindrical, conical, and parabolic etched structures.

## 1. Introduction

Colloidal lithography is a highly promising technique with numerous advantages. It allows for the fabrication of structures ranging from tens of micrometers to several nanometers in size, enabling the creation of periodic micro- and nanostructures. While there are many methods available for the generation of micro- and nano-patterns, such as optical lithography, electron beam lithography, focused ion beam lithography, and nanoimprint lithography, colloidal lithography offers relative simplicity in terms of both the process involved and the equipment required. Moreover, it has already found fascinating applications in various fields. A combination of etching processes can be utilized in biomimetic anti-reflective moth-eye structures, thereby reducing the loss of incident light, and consequently increasing the efficiency of photonic devices. For example, Chan et al. [1] and Chou et al. [2], respectively, fabricated moth-eye structures on the surfaces of LEDs and solar cells to enhance light emission and power generation efficiency. Colloidal particles possess the characteristic of a large ordered surface area. The use of surface-enhanced Raman scattering (SERS) leads to an increase in the scattering cross-section of molecules adsorbed on or near metal nanostructures. This increase in cross-section results in the amplified intensity of the measured Raman signal. Due to the size range for colloidal particles, which can vary from nanometers to tens of micrometers, and their ability to achieve long-range ordered arrangements through templating techniques, even creating micro/nanocomposite 3D structures, they are utilized as low-cost templates for SERS [3,4,5,6]. Colloidal particles can also serve a large number of different carriers, and can find applications in biomedical technology. In their study, L. Liang et al. [7] considered polystyrene (PS) spheres for their specific surface area, good mechanical properties, and high oxygen permeability. They employed Pt(II) octaethylporphyrin (PtOEP) as the oxygen sensing agent, and achieved novel proportional oxygen-sensitive microspheres using a polymerizable methacrylate-derived rhodamine derivative (Rhod-MA). These microspheres were utilized for air pressure measurement, the in situ monitoring of cellular oxygen respiration, and the analysis of dissolved oxygen concentrations in several liquids, including beverages.

To accommodate the various application sizes of colloidal particles, different synthesis methods have been proposed to prepare micro- and nanoparticles of different diameters [8]. Among them, dispersion polymerization is the most commonly employed method. The oil droplet nucleation theory of dispersion polymerization shares many similarities with the homogeneous nucleation theory of emulsion polymerization. However, dispersion polymerization predominantly involves the use of organic solvents, which exhibit a better affinity with the polymer than water. Consequently, the oligomers precipitate out of the solvent with longer chain lengths compared to those precipitating in emulsion polymerization. This results in the microspheres obtained via dispersion polymerization being larger in size than those produced using emulsion polymerization.

To enable the synthesized particles to form stacked arrangements to facilitate subsequent processes, the arrangement of micro/nanospheres has become a crucial technique. J. Chen et al. [9] deposited droplets of spheres onto a substrate, utilizing centrifugal force generated by spin-coating to remove excess droplets, and continued spinning to evaporate the solvent from the central part, ultimately achieving sphere alignment. By adjusting the rotational speed, the spin-coating method can be used to arrange double-layer to multilayer arrangements; however, presenting a densely packed monolayer hexagonal lattice over a large area is more challenging. S. Jeong et al. [10] wound stainless steel wire around a stainless steel rod and then deposited pre-prepared spherical droplets onto the surface of a substrate. The researchers used stainless steel fixtures to roll the substrate, squeezing the microspheres into alignment through the gaps between the stainless steel wires. Microspheres arranged in this manner are prone to forming grain boundaries, stacking, and voids, due to the difficulty in controlling and managing the spacing between the stainless steel wires. E. Sirotkin et al. [11] altered the gas–liquid electrostatic environment to achieve self-assembly alignment. Firstly, hydrophilic microspheres were modified with carboxylic acid to impart a negative charge onto the surface of the microspheres. Sodium ions (Na^+^) were then introduced into the water, utilizing the repulsive force between the modified water and microspheres’ positive and negative charges, enabling the microspheres to remain at the gas–liquid interface. To achieve large-area and single-crystal alignment of microspheres, it is crucial to control the concentration of sodium ions in the water. The findings presented in the literature [11] indicate that increasing the repulsive force between water and microspheres can aid in the avoidance of grain boundary formation.

From the above examples derived from the literature, it is evident that the scope of applications for micro/nanospheres is extensive, as a complete process can be formed through their synthesis, self-assembly alignment, and subsequent application. Dispersed polymerization methods can be used to produce particles of various sizes. In the study presented herein, we explored the impact of synthesis parameters on particle size through the use of synthetic methods. Subsequently, we utilized the floating assembly method to arrange the synthesized nanoparticles into a densely packed monolayer hexagonal lattice. Conventional photolithography technology involves the use of a photoresist on a substrate to define an etching mask layer. However, due to optical limits during exposure, smaller linewidth patterns may suffer from diffraction, resulting in incomplete pattern definition and the resolution being affected. This, in turn, leads to changes in linewidth dimensions during subsequent development processes. In contrast, colloidal lithography is not constrained by optical limitations, allowing for precise pattern size definition. In the present study, we employed colloidal particles as etching masks and, in conjunction with different plasma etching and etching processes, were able to etch out subwavelength cone, as well as cylindrical and parabolic structures.

## 2. Experiment

### 2.1. Synthesis of Micro/Nano PS Spheres

Due to the inherent tendency of styrene monomers to gradually polymerize at room temperature, commercial products are typically supplemented with inhibitors (such as hydroquinone or tertiary-butylcatechol) as stabilizers to delay their polymerization, allowing for storage. To prevent the inhibitors from negatively impacting the experimental results, it is necessary to purify the styrene monomer before conducting experiments. The experiment performed in the present study involved the use of vacuum distillation, utilizing reduced pressure to lower the boiling point and achieve fractionation based on the differing boiling points of components. The boiling points of styrene and hydroquinone are 145 °C and 287 °C, respectively. Under a vacuum environment of 75 Torr, the boiling points of styrene and hydroquinone decrease to 72 °C and 190 °C, respectively. Therefore, the distillation system was operated under a vacuum of 75 Torr and heated to 75 °C to obtain purified styrene through distillation.

The purified styrene monomer was then used to prepare PS spheres via dispersion polymerization. Unlike methods requiring the prior addition of surfactants, dispersion polymerization necessitates achieving a homogeneous phase in the solution before initiating polymerization. The preparation procedure is outlined as follows:

(1) Preparation of the homogeneous solution: Purified styrene monomer, initiator (2,2′-Azobis(2-methylpropionitrile), AIBN), and stabilizer polyvinylpyrrolidone (PVP, K-16) are added to a four-neck flask containing 99.5% ethanol. The mixture is then subjected to ultrasonic agitation to ensure complete dissolution, resulting in a homogeneous solution containing tiny oil droplets, serving as nucleation sites for subsequent processes.
(2) Isothermal polymerization: A silicone oil bath is placed on a heating plate and maintained at 95 °C. The aforementioned four-neck flask is then placed into the oil bath, and the polymerization reaction proceeds for 12 h. Upon completion of polymerization, the solution is allowed to cool naturally for approximately two hours to room temperature.
(3) Particle centrifugation and precipitation: Centrifugal separation is conducted at 9000 rpm for 45 min, resulting in the precipitation of solids. The precipitate is then washed with a mixture of methanol and water to remove any unreacted monomers, initiators, and solvents. Finally, the precipitate is placed in a vacuum oven to allow for drying.

Changing the dosage of the stabilizer, monomer, and initiator will affect the final particle size and distribution. Table 1 presents the process parameters used in this experiment.

### 2.2. Arrangement of Close-Packed PS Spheres

In the following study, the floating assembly method was employed to arrange PS spheres, and automated laboratory equipment for particle alignment was developed. The deposition process is outlined below.

(1) Preparation of the PS Sphere Suspension

The aim of preparing the sphere suspension is to ensure the uniform dispersion of spheres in the liquid. Dry micro/nanoparticle powder is mixed with ethanol and deionized water. The mixture is then sonicated for 1 h using an ultrasonic oscillator to ensure the uniform dispersion of micro/nanospheres in the liquid.

(2) PS Sphere Arrangement
  - Place the silicon wafer on the platform of the lifting module and pour deionized water into the water tank.
  - Draw the prepared sphere suspension into a syringe and place the syringe on the syringe holder of the injection pump module.
  - Set the injection and baffle movement speed, baffle and platform movement distance, and baffle retraction distance.
  - Continuously inject the suspension at the liquid surface. Slowly move the baffle forward until the injection is complete and then move the baffle backward to tightly arrange the PS beads on the liquid surface.
  - Move the lift module upward to detach the substrate from the liquid surface, completing the PS sphere arrangement.

**Figure 1:**
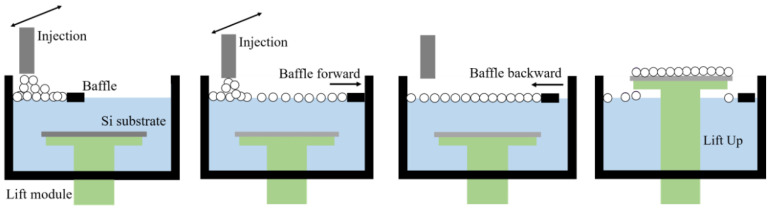
Arrangement of PS spheres using the floating assembly method.

The schematic diagram of the PS spheres arrangement process of D and E is shown in Figure 1. For further details, please refer to ref. [12].

### 2.3. Plasma Etching for Subwavelength Structure Fabrication

**Figure 2:**
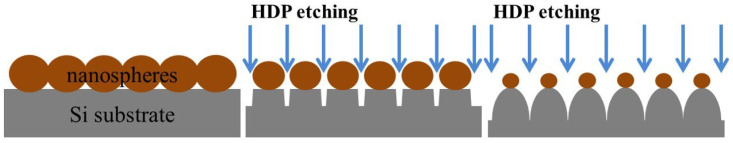
Schematic illustration of the HDP etching processes.

PS sphere deposition on a silicon substrate as etching masks was employed, coupled with a high-density plasma (HDP, Unaxis/Nextral 860L) etching system, to fabricate subwavelength structures. Figure 2 illustrates the proposed fabrication process.

(1) In the dry etching system, the RF power affects the etching rate and surface roughness. In this experiment, 50 W and 100 W were used as the etching powers, respectively.
(2) The gases introduced into the system simultaneously are SF_6_, Ar, and O_2_. SF_6_ is the primary etching gas for the silicon material in this process, with a fixed flow rate of 50 sccm. Ar serves to maintain plasma stability, with a fixed flow rate of 25 sccm. The O_2_ flow rate plays a crucial role in the etching mechanism, as adding a small amount of O_2_ can assist dissociation, increase free radical generation, and enhance the etching rate. However, excessive O_2_ can reduce the etching rate due to dilution effects. Therefore, in the present study, O_2_ flow rates of 15, 20, 35, 40, and 50 sccm were introduced to create various subwavelength structures.
(3) Clean with acetone, isopropanol, and deionized water sequentially for 3 min each, using an ultrasonic cleaner to remove any residual PS spheres.

## 3. Results and Discussion

### 3.1. Parameters Used in Dispersion Polymerization

**Figure 3:**
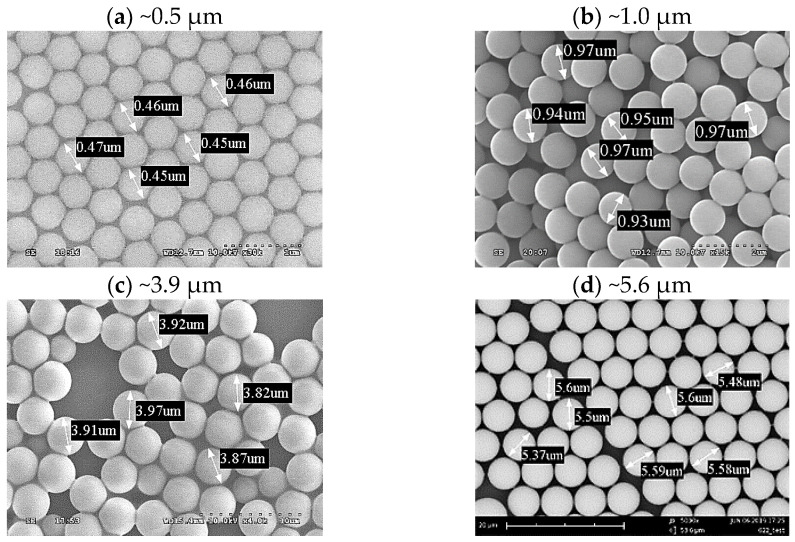
SEM images of PS spheres of different sizes prepared according to the perspective parameters listed in Table 1. For SEM inspection, the spheres are assembled on Si substrate at a temperature of 22 °C from a deionized water–methanol mixture, without attending long-range order.

Figure 3 shows PS spheres of different sizes produced using the parameters listed in Table 1. The results of the experiment demonstrate that PVP can stably control the particle size of the spheres. Naturally, as the size of the spheres increases, more monomer is required, and more initiator is needed to generate additional free radicals.

Monomers, initiators, and stabilizers dissolve uniformly in a solvent to form a homogeneous solution. During polymerization, initiators decompose into primary radicals, which then polymerize with the monomers in the solution. When the polymer chain length exceeds a critical length, oligomeric radicals precipitate from the solvent to form nuclei. These nuclei formed by precipitation are unstable within the system, leading multiple nuclei to aggregate into stable growing microspheres. Stabilizers adsorb onto the surface of the growing microspheres, providing stability. The growing microspheres absorb monomers and radicals from the continuous phase, and undergo polymerization inside the microsphere. At this point, polymerization shifts from the continuous phase to inside the microsphere, continuing until completion [13,14]. Monomer concentration has a significant impact on the growth phase. A sufficient monomer concentration is required for the microspheres to continue growing. The double bonds of the styrene monomer react with the free radicals generated by the initiator, sustaining chain reaction polymerization to form polymers. If the monomer concentration is low, the concentration of the formed chain oligomers will also be low, making it difficult for the polystyrene spheres to absorb and grow, leading to a decrease in average particle size. However, if the monomer concentration is too high, the number of nuclei will be limited, and the microspheres’ absorption rate will not keep pace with the monomer reaction rate. The excess monomers will react to form new nuclei, a phenomenon known as secondary nucleation. This will in turn result in an uneven particle size distribution and excess small spheres in the solution. Therefore, increasing the monomer concentration within an appropriate range can enhance the sphere diameter. The initiator concentration also influences the particle size distribution. When the initiator concentration is too low, the number of primary free radicals decomposed in the system decreases, reducing the rate at which polymer chains precipitate. This process significantly prolongs the nucleation period, resulting in a broader final particle size distribution. Conversely, when the initiator concentration is too high, the growing microspheres are larger, and the rate of formation of oligomer free radicals and inactive polymer chains (i.e., polymer chains that have reached the critical chain length) increases. Consequently, the efficiency of the growing microspheres in capturing these chains decreases. Since the capture efficiency is lower than the formation rate, secondary nucleation may occur within the system, leading to a broader particle size distribution.

In the present study, the stabilizer concentration was primarily controlled to alter the sphere diameter. The concentration of the stabilizer affects the reaction rate of the microspheres and the critical chain length at precipitation, leading to variations in size during the nucleation stage. Since the nucleation and growth stages occur in the continuous phase, the particles are in an unstable state and easily bond with other molecules. Stabilizer molecules, having both hydrophilic and hydrophobic groups, can react with free radicals to form copolymers that adsorb onto the surface of the microspheres. This process forms a protective layer that stabilizes the polymer particles, preventing further reactions or agglomeration. An appropriate amount of stabilizer helps in shaping the spheres and maintaining uniformity. However, adding too much stabilizer can lead to rapid adsorption, hindering the collision and reaction of monomers with the spheres, resulting in a smaller overall particle size and many small spheres that stabilize before reaching full growth. Table 1 shows that by keeping the contents of ethanol, styrene, and AIBN constant, and only reducing the weight of the stabilizer PVP from 40 g to 27 g, the size of the PS spheres increases from 0.5 µm to 1.0 µm. To further obtain PS spheres with a larger diameter, the PVP content was reduced to only 4 g. However, it was also necessary to increase the styrene content to 9 g to provide sufficient monomer, resulting in PS spheres with a diameter close to 4 µm. The use of a lower amount of stabilizer extends the growth phase of the spheres, allowing for continuous monomer adsorption and gradual stabilization. Nonetheless, this prolonged stabilization period can produce many new small spheres, and an insufficient amount of stabilizer can lead to an unstable polymerization system. Some microspheres may struggle to adsorb stabilizer molecules, significantly affecting uniformity. In the last experiment in Table 1, the PVP content was reduced to just 2 g, while the contents of ethanol, styrene, and AIBN were increased. Although the size of the PS spheres increased, the uniformity of the sphere diameters decreased.

### 3.2. Arrangement of PS Spheres

Using the floating assembly method, hydrophobic micro- and nanospheres were injected onto the surface of deionized water and then deposited. The surface tension of deionized water affects the flotation of the spheres on the water surface. Additionally, the composition of the suspension also influences whether the injected spheres stack or disperse on the water surface.

#### 3.2.1. Effect of Surface Tension on the Arrangement of PS Spheres

**Figure 4:**
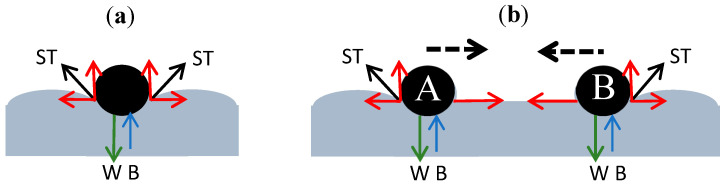
(**a**) Schematic diagram of one particle with vertical and horizontal components of surface supporting force in balance; (**b**) schematic diagram of particles A and B approaching each other due to imbalance in horizontal components of surface supporting force.

For polystyrene microspheres, there exists a hydrophobic characteristic between them and water. The vertical component of the surface tension force (ST) balances with gravity (W) and buoyancy (B), while the horizontal component balances in the horizontal direction, as shown in Figure 4a. When hydrophobic microspheres approach one another, the convex water surface between the two microspheres tends to level out due to surface tension. This causes the surface tension to become horizontal, disrupting the balance of the horizontal components. The resultant horizontal force on microsphere A points to the right, and the resultant horizontal force on microsphere B points to the left, ultimately causing microspheres A and B to move closer to each other due to the direction of the resultant forces, as shown in Figure 4b.

**Figure 5:**
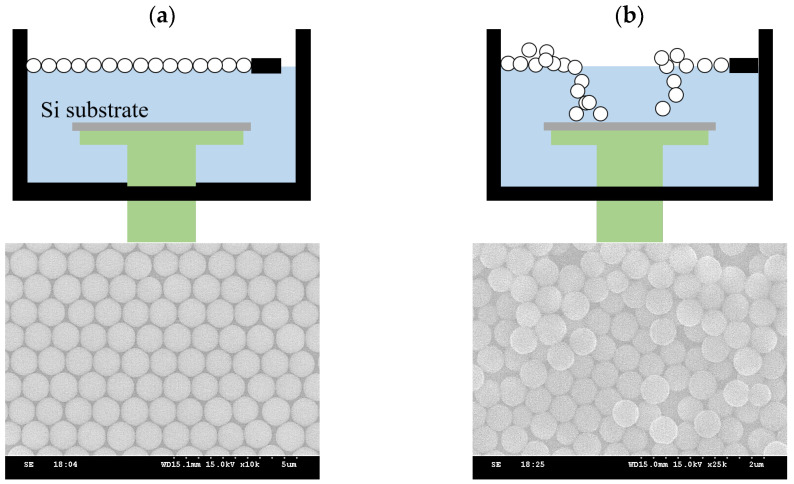
Results of PS sphere arrangement in deionized water at different temperatures, as follows: (**a**) 20 °C and (**b**) 35 °C.

Factors affecting surface tension include pressure, liquid purity, and temperature [15]. Surface tension decreases with increasing temperature. As the temperature of the liquid rises, the momentum of the liquid molecules increases, reducing the mutual attractive forces between them. This process then leads to a decrease in the attractive forces pulling the liquid molecules within the surface layer toward the interior of the liquid, ultimately resulting in a reduction in surface tension. We achieved PS sphere arrangements using deionized water at two temperatures, 20 °C and 35 °C. At the lower temperature, the higher surface tension allowed the spheres to effectively float on the water surface, resulting in a closed arrangement. At the higher temperature, some spheres partially sunk into the water, while others floated on the surface but in a stacked form. This then led to the deposition process failing to achieve a monolayer hexagonal lattice arrangement. The schematic diagram and SEM images are shown in Figure 5.

#### 3.2.2. Modification of Repulsive and Attractive Forces

**Figure 6:**
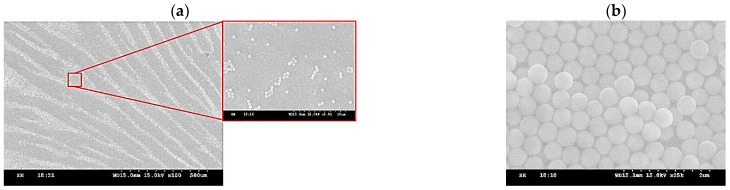
Arrangement of PS spheres on Si from a suspension in deionized water and (**a**) methanol, (**b**) ethanol, obtained at 22 °C.

The hydrophobic PS spheres synthesized in the experiment, described herein as colloidal particles, do not carry surface charges. Nevertheless, the sphere suspension consists of PS spheres, deionized water, and either methanol or ethanol. In our experiment, we fixed the ratio of PS spheres to deionized water and adjusted the amounts of methanol and ethanol. Figure 6 shows the results of spreading the spheres with a ratio of methanol (ethanol)–PS spheres–deionized water = 1:2:2. From the results, it can be observed that using methanol causes the injected microspheres to be more dispersed on the liquid surface, resulting in the deposited PS spheres being unable to densely arrange, and appearing irregularly dispersed, as shown in Figure 6a. Conversely, using ethanol causes the injected microspheres to aggregate and stack more closely on the liquid surface, as shown in Figure 6b. The surface tensions of methanol/air and ethanol/air at 20 °C are 22.50 mN/m and 22.39 mN/m, respectively, showing a negligible difference. However, Figure 6 illustrates significant differences in suspensions of methanol and ethanol mixed with PS powder and deionized water at the same proportions. This result may be explained by methanol having higher polarity than ethanol, resulting in stronger Coulomb repulsive forces generated by the surface charge of the particles in the methanol.

As the distance between two particles decreases, they experience van der Waals forces, leading to mutual attraction and the formation of a closely packed arrangement. This attraction is inversely proportional to the distance between particles, and is known as the induction dipole–induced dipole interaction within van der Waals forces, also referred to as London dispersion forces. Therefore, colloidal particles may be mutually attracted, leading to a closely packed arrangement. During the arrangement process of PS particles, appropriate baffles backward, applying external force to bring the microspheres closer together to facilitate self-assembly arrangement. The agitation from external forces causes water inside the container to ripple. Using the water’s motion, the distance between the PS spheres around the void areas is reduced, allowing hydrophobic spheres to approach each other and be attracted, resulting in arrangement formation.

**Figure 7:**
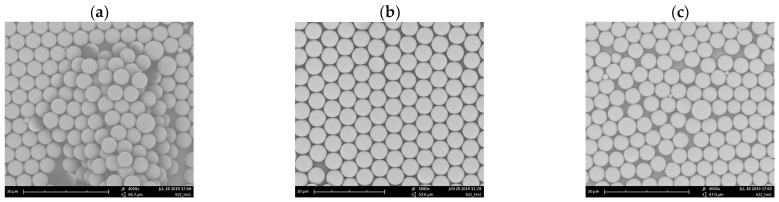
Arrangement of PS spheres at different ratios of ethanol to PS spheres to deionized water. (**a**) 1:2:2; (**b**) 2:2:2; (**c**) 3:2:2.

Due to the tendency of methanol solution to induce the dispersion of PS spheres, ethanol solution was adopted in the experiment described herein, with adjustments made to the ethanol ratio. Figure 7 shows the arrangement results for ethanol–PS spheres–deionized water ratios of 1:2:2, 2:2:2, and 3:2:2. As the ethanol concentration increases, the dispersion between PS spheres improves. When the ratio is 2:2:2, a hexagonal, closely packed arrangement is achieved.

### 3.3. Plasma Etching for Subwavelength Structure

HDP etching, characterized by its high dissociative ion density, offers faster etching rates, better etching selectivity, and superior sidewall profile control [16]. The aim of the present study was to fabricate subwavelength structures while simultaneously considering both vertical and lateral etching capabilities. Using 0.5 μm PS spheres as etching masks, etching capability was enhanced by increasing the RF power, and the morphology of subwavelength structures was adjusted by varying the O_2_ flow rate within the vacuum chamber.

RF power levels of 50 W and 100 W were employed for etching durations of 2 min and 5 min, respectively. The experimental results are shown in Table 2. Under the conditions of RF power of 50 W for 2 min, etching occurs through the gaps between PS spheres, resulting in flat-topped conical structures with edges. Similar cone-shaped structural profiles were obtained for both parameter sets, as follows: RF power of 100 W for 2 min of etching and RF power of 50 W for 5 min of etching. The etching rate with an RF power of 100 W was faster compared to that achieved with an RF power of 50 W, and stronger oxygen plasma etching capabilities were also exhibited to remove PS spheres. Prolonging the etching time may lead to excessive etching, resulting in the structures not retaining the desired geometry and height.

**Figure 8:**
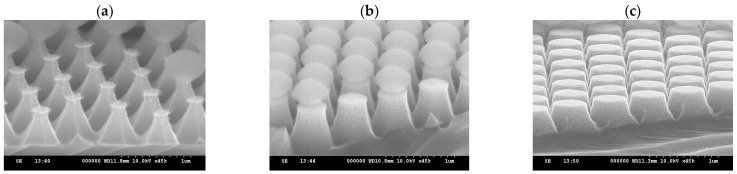
SEM images of etching for 5 min with fixed RF power at 50 W and chamber pressure at 25 mTorr; the gas flow rates of SF_6_ and Ar were kept constant at 50 sccm and 25 sccm, with different O_2_ flow rates of (**a**) 5 sccm, (**b**) 15 sccm, and (**c**) 25 sccm.

In the HDP system, the gases introduced were SF_6_, Ar, and O_2_. SF_6_ is the primary gas used for etching silicon material in this process. Ar maintains plasma stability, while O_2_ plays a crucial role in the etching mechanism. In a plasma environment, SF_6_ generates fluorine radicals to etch silicon and form SiF_4_. Subsequently, SiF_4_ reacts with oxygen radicals to form SiOF, creating a passivation of the silicon surface, and thereby causing anisotropic etching [17,18]. To observe the above trend, in our experiment, an RF power of 50 W was selected to compare different O_2_ flow rates and observe their effects on etching rate and structural morphology, respectively. Different O_2_ flow rates of 5, 15, and 25 sccm were introduced, and the etching process was conducted for 5 min. Figure 8 shows the SEM images obtained after adjusting the O_2_ flow rate individually. Observing Figure 8a,b, the vertical etching rates are similar. However, due to the lower oxygen content in Figure 8a, the lateral etching capability of Si is smaller compared to Figure 8b, resulting in a faster lateral etching rate. Furthermore, a smaller O_2_ gas flow results in isotropic etching of Si. When the oxygen flow rate increases again in Figure 8c, the lateral etching capability becomes worse, and the vertical etching capability is also affected. With RF power = 50 W, the oxygen cannot be sufficiently dissociated when the oxygen flow increases from 15 sccm to 25 sccm, so the PS sphere itself remains more intact. Under the same etching conditions, the flat-top area of the structure in Figure 8c is larger.

**Figure 9:**
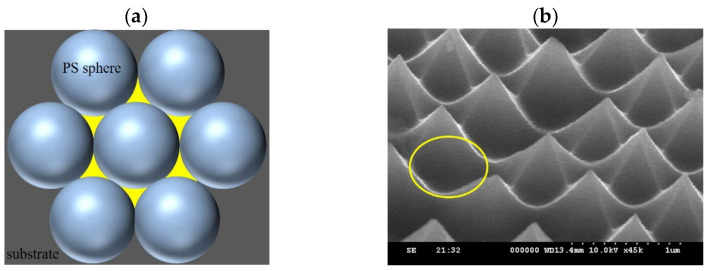
(**a**) Schematic diagram of the arrangement gaps between PS spheres and the (**b**) SEM image of the etching structure. The yellow area on the left shows the gaps between the tightly packed PS spheres, which are the regions prone to etching. The etching produces the indentations circled in yellow on the right.

With the same etching duration and an O_2_ flow rate of 5 sccm, it was inferred that there was a higher etching rate on the substrate, indicating stronger lateral etching capability. Analysis of Figure 9 reveals that, due to the gaps between the PS spheres (highlighted in yellow), plasma etching can freely penetrate between the PS spheres, resulting in the formation of conical structures with six-sided edges. As the O_2_ flow rate increases, the etching gas concentration decreases, leading to a reduction in the silicon etching rate. Consequently, the lateral etching capability decreases, resulting in the structural profile approaching a columnar shape. Additionally, the results of previous studies [17,18] have indicated that oxygen plasma can effectively reduce the diameter of PS nanospheres. This process increases the gaps between adjacent PS spheres, allowing for uniform penetration of the plasma etching. As a result, the sidewalls of the structure do not develop edges.

**Figure 10:**
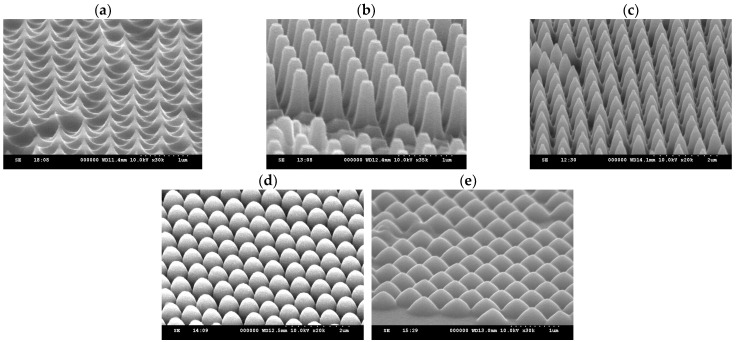
SEM images of etching for 5 min with fixed RF power at 100 W and chamber pressure at 25 mTorr; the gas flow rates of SF_6_ and Ar were kept constant at 50 sccm and 25 sccm, with different O_2_ flow rates of (**a**) 15, (**b**) 25, (**c**) 35, (**d**) 40, and (**e**) 50 sccm.

Based on the experiments described above, it can be observed that increasing the RF power enhances silicon etching capability; in comparison, increasing the O_2_ flow rate reduces silicon etching capability, but enables further etching of the PS spheres, resulting in structures without edges. In light of the above, we adjusted the RF power back to the value of 100 W and introduced O_2_ flow rates of 15, 20, 35, 40, and 50 sccm for 5 min of etching, while keeping the other gas parameters unchanged. The etching results are shown in Figure 10. At an RF power of 100 W, the ability of the etching gas to dissociate into radicals is excellent; however, O_2_ at 15 sccm is insufficient to form a complete passivation, resulting in strong etching of the silicon, forming a conical structure with edges, as shown in Figure 10a. When the O_2_ flow rate is increased to 25 sccm, the SiOF passivation, as previously mentioned, again reduces the sidewall etching ability of the silicon, resulting in a steeper profile, as shown in Figure 10b. As the O_2_ flow rate continues to increase, it will significantly erode the PS spheres due to the oxygen plasma, causing the protective ability of the etching mask to disappear. Consequently, the height of the etched parabolic structures is significantly reduced, as shown in Figure 10d,e.

## 4. Conclusions

The following study primarily involved investigating the synthesis of polystyrene micro/nanospheres, their close packing arrangement, and the fabrication of subwavelength cone structures through plasma etching. The conclusions derived are as follows:

(1) Preparation of submicron- to micron-sized polystyrene microspheres through dispersion polymerization: The appropriate amount of stabilizer can aid in the formation of PS spheres and maintain a certain level of uniformity. Adding an excessive amount of stabilizer can lead to overly rapid adsorption, making it difficult for monomers to react with the microspheres upon collision, resulting in an overall decrease in particle size.
(2) Using the floating assembly method to arrange PS spheres: Surface tension effectively enables the microspheres to form a monolayer arrangement on the liquid surface, while van der Waals forces and Coulomb repulsion maintain a static equilibrium, allowing the particles to maintain a fixed distance of arrangement. Disturbing the floating system with external forces can effectively promote the tight arrangement of PS spheres.
(3) The fabrication of subwavelength cone structures can be achieved using HDP etching, where the control of oxygen flow significantly impacts the morphology of the subwavelength structures.

## Figures and Tables

**Table 1:** The weight of the solvent (ethanol), monomer, initiator, and stabilizer used in dispersion polymerization.

Exp.	Ethanol (g)	Styrene (g)	AIBN (g)	PVP (g)
(a)	47.5	6	0.18	40
(b)	47.5	6	0.18	27
(c)	47.5	9	0.18	4
(d)	67.2	30	0.8	2

**Table 2:** SEM images of etching with different RF powers and etching times, with the same gas flow rates of SF6: 50 sccm, Ar: 25 sccm, O_2_: 5 sccm, and chamber pressure: 25 mTorr.

E/T (min)	RF Power (W)	
	50	100
2	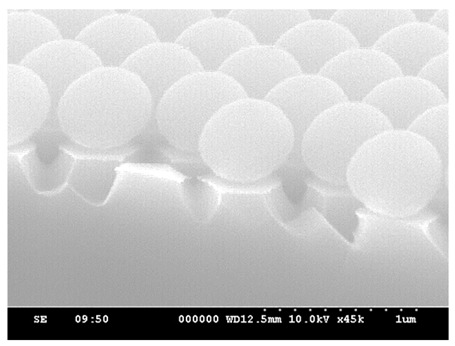	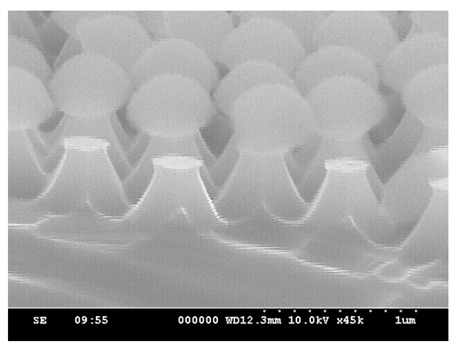
5	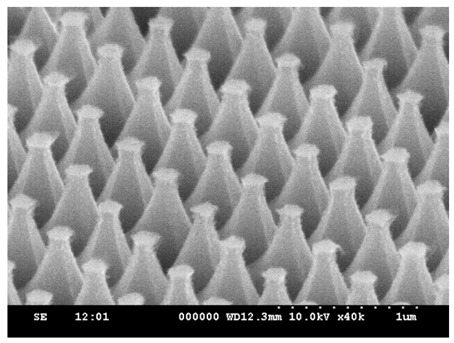	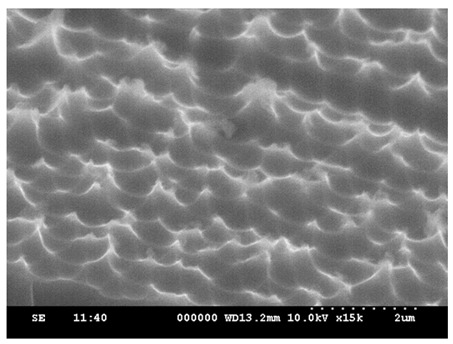

## Data Availability

Data are contained within the article.
